# Implementation of an integrated care strategy for child contacts of tuberculosis patients: a quasi-experimental study protocol

**DOI:** 10.1186/s12887-022-03798-x

**Published:** 2023-01-19

**Authors:** Dione Benjumea-Bedoya, Esteban Villegas Arbeláez, Daniela Martínez-Peñaloza, Claudia Patricia Beltrán-Arroyave, Andrea Victoria Restrepo Gouzy, Diana Marín, Lina Marcela Cadavid Álvarez, Beatriz Molinares, Fernando Nicolás Montes Zuluaga, Henry Pulido Duarte, Pedro Mauricio Suárez Parra, Juan Carlos Alzate Ángel, Guillermo Vélez-Parra, Teresa Realpe, Marisol Vásquez Villa, Stefani Yamile Ríos Buitrago, Jenifer Puerta Zapata, Lisandra María Arango García, Yesenia Perea Torres, Natalia Pérez Doncel, María Patricia Arbeláez Montoya, Jaime Robledo

**Affiliations:** 1 Unidad de Bacteriología y Micobacterias, Corporación para Investigaciones Biológicas, Universidad Pontificia Bolivariana, Medellín, Colombia; 2 Grupo de Investigación en Salud Familiar y Comunitaria, School of Health Sciences, Corporación Universitaria Remington, Medellín, Colombia; 3 Grupo de Epidemiología, Facultad Nacional de Salud Pública, Universidad de Antioquia, Medellín, Colombia; 4 Grupo Pediaciencias, Medicine School, Universidad de Antioquia, Medellín, Colombia; 5 Clínica El Rosario, Medellín, Colombia; 6 Clínica del Prado, Medellín, Colombia; 7 Hospital Pablo Tobón Uribe, Medellín, Colombia; 8 Grupo de Investigación en Salud Pública, School of Health Sciences, Universidad Pontificia Bolivariana, Medellín, Colombia; 9 Tuberculosis Program, Secretariat of Health of Medellín, Medellín, Colombia; 10 Tuberculosis Program, Secretariat of Health of Bello, Bello, Colombia; 11 Tuberculosis Program, Secretariat of Health of Itagüí, Itagüí, Colombia; 12 Hospital General de Medellín, Professor Universidad CES, Medellín, Colombia; 13 School of Health Sciences, Universidad Pontificia Bolivariana, Medellín, Colombia

**Keywords:** Latent Tuberculosis, Comprehensive Health Care, Person-Centered Care, Child, Preschool, Rifampin

## Abstract

**Background:**

Childhood tuberculosis continues to be a major public health problem. Although the visibility of the epidemic in this population group has increased, further research is needed.

**Objective:**

To design, implement and evaluate an integrated care strategy for children under five years old who are household contacts of bacteriologically confirmed pulmonary tuberculosis patients in Medellín and the Metropolitan Area.

**Methods:**

A quasi-experimental study in which approximately 300 children who are household contacts of bacteriologically confirmed pulmonary tuberculosis patients from Medellín and the Metropolitan Area will be evaluated and recruited over one year. A subgroup of these children, estimated at 85, who require treatment for latent tuberculosis, will receive an integrated care strategy that includes: some modifications of the current standardized scheme in Colombia, with rifampicin treatment daily for four months, follow-up under the project scheme with nursing personnel, general practitioners, specialists, professionals from other disciplines such as social work, psychology, and nutritionist. Additionally, transportation and food assistance will be provided to encourage treatment compliance. This strategy will be compared with isoniazid treatment received by a cohort of children between 2015 and 2018 following the standardized scheme in the country. The study was approved by the CIB Research Ethics Committee and UPB. ClinicalTrials.gov identifier NCT04331262.

**Discussion:**

This study is expected to contribute to the development of integrated care strategies for the treatment of latent tuberculosis in children. The results will have a direct impact on the management of childhood tuberculosis contributing to achieving the goals proposed by the World Health Organization's End TB Strategy.

**Trial registration:**

ClinicalTrials.gov identifier NCT04331262. Implementation of an Integrated Care Strategy for Children Contacts of Patients with Tuberculosis. Registered 2 April 2020.

**Supplementary Information:**

The online version contains supplementary material available at 10.1186/s12887-022-03798-x.

## Background

Tuberculosis (TB) in childhood is considered a public health emergency, particularly in developing countries [1], where it accounts for approximately 15% of TB cases [2, 3]. This form of the disease remains relatively ignored, mainly because of the major diagnostic challenges and low priority given by TB control programs. In 2012 the WHO began to publish data on the incidence of TB in children under 15 years of age separated from adults, showing the need to give more importance to childhood TB [4].

Compared to adults, children respond differently to the disease. This has important implications for prevention, diagnosis, and treatment. In addition, children living in close contact with bacteriologically confirmed pulmonary TB patients are at particular risk of infection (50%) and TB disease (8.5%), and progression from primary *M. tuberculosis* infection to disease, especially infants and children under five years of age. This makes them a target group for the treatment of latent TB infection (LTBI). Likewise, much of the postprimary TB is due to cases of reactivation of childhood-acquired infections [3, 5–12]. However, children who are household contacts of pulmonary TB patients do not receive proper care in the national TB control programs [3]. In 2020, the WHO reported that in Colombia, 38% of children under five years who were in household contact with bacteriologically confirmed TB patients were on treatment for LTBI [13].

The WHO recognizes the importance of identifying the best strategies to trace contacts, especially in children, and researching the best-tolerated TB preventive treatment regimens compared to those currently recommended [14, 15]. This highlights the need for studies on the identification of strategies to improve adherence to TB preventive treatment in children, the reporting and management of child contacts, identification of barriers reports including the private sector, access to health services and integrated services, and implementation/operational research on the use and impact of the newly developed integrated treatment decision algorithms [15].

The best-studied regimen for LTBI treatment is isoniazid 10 mg/kg per day, not exceeding 300 mg per day, in single doses for 12 months [16]. This regimen is almost 100% effective in children and protects them for at least 30 years [16, 17]. Most authorities recommend at least nine months of preventive treatment in children [16], but at least six months of continuous medication appears to be necessary to achieve a significant reduction in the risk of developing TB [18].

The 2020 WHO LTBI treatment guidelines recommend other treatment regimens with shorter duration and equal efficacy even for children, which could favor higher treatment completion rates [19]. These include rifampicin for four months and rifampicin plus isoniazid for three months. This scheme is already being used in some countries [20, 21]. However, the current recommendation for the treatment of latent TB in Colombia is still isoniazid, and recently, a scheme of 12 weekly doses of isoniazid and rifapentine exclusively for patients with HIV infection has been implemented [22].

To achieve the goals set by the WHO in its "End TB" strategy [23], it is necessary not only to fight active TB but also to address LTBI with effective strategies. Therefore, research on LTBI is also needed. Important aspects that have been identified comprise improvement of diagnostic tests and test performance for LTBI, shorter and better-tolerated treatment options, the study of efficacy and side effects of some drugs, especially in children, duration of protection of preventive treatments in high prevalence conditions, the benefit of liver enzyme monitoring, adherence and treatment completion, evidence of the effectiveness of context-specific interventions to improve adherence and treatment completion depending on the availability of resources, health system infrastructure, the use of "digital health", cost-effectiveness studies of LTBI treatment, programmatic management, the burden of LTBI in different at-risk populations, including community-based approaches, implementation of home-based models, the development of tools to facilitate the monitoring and evaluation of programmatic management of LTBI [19].

Therefore, it is necessary to propose an intervention strategy for the integrated care of children who are contacts of pulmonary TB patients. This would allow quick identification of children with active TB and timely treatment for LTBI as a preventive strategy for active TB [19].

This study will show the capability of a new comprehensive care strategy to prevent the development of TB in children who are contacts of bacteriologically confirmed pulmonary tuberculosis patients. The new strategy includes patient-centered care, based on primary health care, with a multidisciplinary approach, a less extensive treatment scheme, and incentives to favor LTBI treatment compliance and completion. The data gathered in this study can contribute to updating the current TB guidelines in Colombia and support updated WHO guidelines with stronger evidence.

### Study objective

To design, implement and evaluate an integrated care strategy for children under five years old who are household contacts of bacteriologically confirmed pulmonary tuberculosis patients in Medellín and the Metropolitan Area.

## Methods

### Study design

This quasi-experimental study will compare the outcomes of the implementation of two treatment strategies for LTBI in children in Medellín and the Metropolitan Area (Valle de Aburrá), Antioquia, Colombia. The intervention cohort will receive an integrated care strategy, which consists of diagnostic tests, the supply of a scheme treatment with daily rifampicin for four months, active monitoring, permanent education and contact with the parents and/or guardians. In addition, social, psychological, and nutritional assistance will be provided, with a 12-month follow-up. The control group will be the historic 2015 to 2018 cohort who received the standard care protocol, with a 9-month scheme of isoniazid [24].

This protocol was conceived following the Standard Protocol Items: Recommendations for Interventional Trials (SPIRIT) guidelines [25] (supplement 1).

### Study setting

The children will be clinically assessed, at first, at *Corporación para Investigaciones Biológicas* (CIB), a research and healthcare facility located in Medellín, in a consulting room adapted for clinical and epidemiological purposes. The diagnosis of active TB will be ruled out, and the diagnosis of LTBI will be confirmed. Treatment initiation and monthly clinical monitoring of treatment adherence, and possible side effects will be conducted at the same location. Similarly, the immune response assessment (tuberculin skin test-TST, and Interferon Gamma Release Assay-IGRA), blood samples, social, psychological, and nutritional evaluation of the participants as well as the 12-month clinical visit after the initial assessment will be carried out at the same facility.

Chest radiography (including standardized reading) and liver function analysis will be performed at the *Pablo Tobón Uribe Hospital*; when active TB needs to be ruled out, microbiology samples (induced sputum and gastric juice aspirate) will be obtained at the *Hospital Infantil Concejo de Medellín*. These samples will be tested with smear, culture, and Xpert MTB/RIF Ultra.

### Study population

The population will be the children under five years who are household contacts of bacteriologically confirmed pulmonary TB patients from Medellín and the Metropolitan Area, notified to the surveillance system during 2021 and 2022, for whom LTBI treatment is indicated. The sampling method is nonprobabilistic since TB household contact children will be recruited as new cases (incidents) of smear-positive confirmed pulmonary TB appear in Medellín and the Metropolitan Area.

### Sample size

According to previous studies, it is estimated that approximately 250 to 300 children who are household contacts of bacteriologically confirmed pulmonary TB patients from Medellín and the Metropolitan Area will be assessed for one year, with an estimated proportion of infection of 73.5%. The complete intervention of the integrated care strategy (with medication, incentives, and active follow-up) will be provided to children who require treatment for LTBI and who will also be selected incidentally, according to their willingness to participate. Considering the 59% isoniazid treatment adherence in the 2015–2018 cohort, adherence with the proposed strategy around 80%, 95% confidence, and 80% power, it is estimated that 75 children will be required in the proposed comprehensive care strategy (Epidat V 4.2). An additional 10% will be estimated due to lost to follow-up, therefore the total number of children to enter the integrated care strategy will be 85.

### Eligibility criteria

For the initial evaluation, we will include all children under five years, who are household contacts of a bacteriologically confirmed pulmonary TB patient, live in Medellín or the Metropolitan Area, have a TST response ≥ 5 mm or an IGRA (QuantiFERON®-TB Gold Plus—QFT Plus) positive, are asymptomatic and without clinical signs of active TB, have a normal chest X-ray, and the parents or legal guardian sign the informed consent. Additionally, those children who are in the immunological window period, which refers to the time between potential infection and when a test (i.e., TST or IGRA) can accurately be positive for the infection, will also be included. In this specific scenario, the immunological window period comprises two months from the last exposure to the patient, that is, two weeks after treatment initiation. Children who have contraindications of receiving rifampicin have symptoms or signs of active TB and that the disease has not been ruled out, a liver disorder background, contraindication for performing induced sputum, severe asthma and/or plans to move out to a city out of the study coverage area will be excluded. The study is not gender-based, and healthy volunteers are not accepted.

### Intervention

The intervention will be the integrated care strategy, in which the effects of interventions established in the regulations of the TB program at the national level will be observed, such as the diagnosis and administration of treatment for LTBI in children who are household contacts of pulmonary TB patients. with three main modifications: the supply of daily oral rifampicin (oral suspension) in a four-month self-administered scheme, incentives such as transport and food assistance and the multidisciplinary support. In addition, immune response tests will be performed to measure interferon-gamma production levels (QuantiFERON®-TB Gold Plus—QFT Plus test). This strategy should allow children to be assessed at a single site avoiding going through different institutions and levels of authorization. Integrated care should include a general practitioner, a pediatrician, specialists such as a child infectious disease specialist, patient-centered care, and multipurpose visits. All children will be monitored for 1 year follow-up.

### Standard of care

The control group will be the historic 2015 to 2018 cohort of children with a diagnosis of LTBI who received a nine-month self-administered daily oral isoniazid scheme. The isoniazid comes in tablet form and must be diluted in water to be taken.

### Procedures

The standardization of the personnel who will participate in the study will be carried out in data collection, clinical assessment, and information processing. The procedures will be checked monthly, for quality purposes.

#### Children recruitment

Children will be recruited through contact with the TB control program of Medellín health authorities and other municipalities in the Metropolitan Area that agreed to participate. They will immediately supply the information on children under five years who are household contacts of pulmonary TB patients recently diagnosed and reported to the program. Health authorities’ staff will request authorization from children’s parents or guardians to share information with the research team and support in socializing the project to promote children's participation.

A nurse assistant (research staff) will collect data such as children's age, contact with a bacteriologically confirmed TB patient and the location by telephone contact. Additional information about the children and TB patients will also be collected at this moment. If the child's guardian is willing to participate in the study, an appointment is scheduled. Written informed consent signed by children´s parents or guardians will be required before any initial activities of the study.

#### First meeting

A general practitioner will perform a basic clinical assessment, including a complete interview and physical examination with emphasis on signs of active TB and the verification of the eligibility criteria. A trained health provider will apply the TST and obtain a blood sample (5 ml of venous blood) for the evaluation of interferon-gamma production (QuantiFERON®-TB Gold Plus-QFT Plus test). A TST reading will be performed after 72 h. Then, a chest X-ray will be performed to rule out the active disease with a standardized reading (two independent readers, with a standard form to fill in the information, and if there is disagreement, a third reader is involved to solve the discrepancy). Children with criteria for LTBI treatment will be invited to participate in the integrated care strategy. If the children’s parents or legal guardians do not want to continue the process, they will be referred to the primary healthcare facility within the health system.

#### Integrated care strategy

To guarantee treatment completion for LTBI with rifampicin for 4 months, the study will perform a strategy including six in-person assessments. The first five assessments are monthly, and the last one is in the twelfth month after recruitment. Additionally, two phone contacts are included, one 15 days after the beginning of the strategy and one at the eighth month. If needed, extra assessments could be scheduled. On the first assessment, the children will start the integrated care strategy, and they will receive care from a nursing assistant, a general practitioner, a pediatrician as needed, a nutritionist, a psychologist, and a social worker. The nutritionist assesses the child's nutritional status, anthropometric evaluation, eating habits and behaviors. Counseling is provided at the most convenient times to receive the medication and recommendations on fasting times. The psychologist performs a general assessment of the child's development, as well as an interview about family dynamics and parenting patterns to identify potential barriers or facilitators for parents or caregivers to adhere to the treatment scheme. The social worker will evaluate the children’s social, economic, housing conditions and family environment, as well as the social support network and other conditions that could lead to poor adherence to the therapeutic scheme. The social worker will also guide on government assistance routes in case situations of social vulnerability are identified. In this first meeting, the initial doses of rifampicin suspension are delivered, together with transport and food assistance. If at any point in the strategy, signs of physical or phycological abuse appear, the route of action stipulated by the Colombian authorities will be activated. The parents or legal guardians are instructed in the correct administration of the treatment, along with possible side effects. Then, at the monthly assessment, the general practitioner will evaluate the participants, recalculate the rifampicin dosage, supply the next doses, assess the adherence and side effects, and identify signs and symptoms of active TB. Liver function tests will be conducted during the second month of treatment. If during the in-person assessments or phone contacts, any child might have clinical or radiological criteria for active TB, an additional assessment will be scheduled, if indicated, induced sputum (two samples), aspirating gastric juice (two samples) and a single stool sample will be obtained. These samples will be processed for smear, liquid culture and Xpert MTB/RIF Ultra. In case of positivity for any of the samples, an evaluation for a pediatric infectious disease specialist will be performed, and the child will be referred to a primary healthcare facility to receive treatment for active TB. A private phone line will be available for the study to facilitate permanent communication with the parents or legal guardians and monthly monitoring. If any difficulty that could compromise treatment adherence is identified, the research staff (pediatrician, nutritionist, social worker, and psychologist) will perform additional assessments.

Unless it is strictly necessary or medically indicated, the parents or legal guardians will be exhorted to not give other medication or supplements to the children until the finalization of the four-month treatment scheme.

For children in the immunological window who started rifampicin treatment, TST will be performed two months later; if the result is still negative, treatment can be stopped according to clinical assessment. If TST turns positive, the fourth month of treatment should be completed.

### Outcomes

The primary outcome is treatment compliance, defined as the proportion of children who completed the treatment.

The secondary outcome is adverse events, defined as the proportion of children who presented any reaction during treatment.

### Operational hypothesis

The proportion of treatment compliance for latent TB in contact children with bacteriologically confirmed pulmonary TB patients in Medellín and the Metropolitan Area who receive the integrated care strategy is greater than the proportion of treatment compliance for children who received isoniazid in the 2015–2018 cohort.

### Data collection

Data are collected in a Microsoft Access application designed for the study, which is located on a secure server of the institution, with a personal identification number and a password for each member of the staff. The anthropometric measurements are collected in Anthro Software. The staff has a standardized procedure to collect the information. Every day, a staff member is responsible for double-checking the integrity and consistency of the data collected.

### Data analysis

To describe the characteristics of children's exposure to TB, a univariate analysis will be conducted, this will include the variables corresponding to the index case, the child's exposure, and other epidemiological variables.

Frequency distributions and proportions will be estimated to describe qualitative variables such as sex, socioeconomic stratum, affiliation to the health system, history of BCG vaccination, bacteriological study, and other variables.

The characteristics of the children in the 2015–2018 and 2020–2022 cohorts will be compared using the Z test for the difference in proportions for the qualitative variables and using the Mann‒Whitney U test for the quantitative variables according to the normality of those variables estimated with the Shapiro Wilk test. The characteristics of the index cases and children will be analyzed separately.

To examine the cellular immune response to *M. tuberculosis*, a univariate analysis will be performed. The proportion of response to the TST will be calculated according to categories in < 5 mm, between 5 and 9 mm and ≥ 10 mm, and the prevalence of positive response with a cut-off point ≥ 5 mm. The proportion of interferon-gamma production (QuantiFERON®-TB Gold Plus—QFT Plus) will be calculated according to the categories of positive and negative results.

The total agreement between the two immunological tests will be analyzed, as well as the different possibilities of discordance. The kappa index will also be calculated. The prevalence ratio of TST ≥ 5 mm and/or positive QFT will be calculated, and a bivariate analysis will be made with the characteristics of exposure to TB in children, adjusting for the index case cluster using Poisson regression. Subsequently, a multivariate analysis will be performed adjusting for the variables that met the Hosmer‒Lemeshow criterion in the bivariate analysis (*p*-value < 0.25).

A description of the latent TB treatment characteristics with a univariate analysis estimating proportions of qualitative variables related to administration of the treatment, side effects and treatment compliance (temporary or permanent suspension, cause of treatment suspension) will be made.

The difference in treatment compliance proportions between the 2015–2018 and 2020–2022 cohorts will be calculated, with 95% confidence.

The study timeline is outlined in Table 1.

**Table 1:** Study timeline of enrolment, interventions, and assessments

Research activities	Study period							
	**Year 1**				**Year 2**			
	**Q1**	**Q2**	**Q3**	**Q4**	**Q1**	**Q2**	**Q3**	**Q4**
Training of study staff	X							
**Enrolment**								
Eligibility screen		X	X	X	X			
Informed consent		X	X	X	X			
Clinical assessment		X	X	X	X			
Diagnostic tests		X	X	X	X			
**Interventions**								
Multidisciplinary assessment		X	X	X	X			
Treatment initiation		X	X	X	X			
Treatment follow-up		X	X	X	X	X		
**Assessments**								
Primary outcome: treatment compliance		X	X	X	X	X		
Secondary outcome: adverse events		X	X	X	X	X		
Epidemiological, clinical, and diagnostic tests analysis						X	X	
End of follow-up						X	X	X
Final report							X	X

*Q* Quarter of a year

## Discussion

Among contacts of people with TB under the age of five, 8.5% have active TB, and more than 50% of household contacts have LTBI [3, 6, 8–12]. To face this situation, the WHO calls for improving the detection of children with latent and active TB and for studies in epidemiology, basic research, development of new diagnostic tools, drugs and vaccines, operational, and health systems and services research, as well as the definition of strategies to rule out active TB in children exposed to TB [19, 26–28].

Furthermore, several conditions are related to the effectiveness of treatment for LTBI in children. One of them is adherence to treatment, which in turn is influenced by the behavior followed when providing treatment to an asymptomatic child [29], the occurrence of adverse effects [30], and clinical follow-up during treatment [31]. Additionally, social aspects such as migration, access to health services [18], the perception and knowledge about TB of parents and caregivers, as well as health care providers [32–36]. Similarly, the characteristics of *M. tuberculosis* exposure, history of BCG vaccination [37], clinical and paraclinical evaluation to rule out active TB and to diagnose LTBI before treatment initiation [38] should also be considered when evaluating the effectiveness of treatment for LTBI in children. The study of these issues using qualitative techniques has gained momentum in recent years [39–42]. However, these types of studies are still missing in Latin America.

The present protocol study was proposed to explore possible solutions to issues reported by previous studies related to children exposed to TB in the same setting. One of the previous research projects was a cohort study conducted in Medellín, Colombia between 2005 and 2009, finding a TB incidence rate of 3.4% in children under five years living with patients with pulmonary TB. Of the total active TB detected in household contacts who were followed up, 21.6% corresponded to cases detected in children under five years of age [43]. Despite the high risk of developing active TB in these children and the fact that international guidelines recommend offering treatment for LTBI, in Colombia, the regulations at that time indicated LTBI treatment only for children under five years, not vaccinated with BCG and with a positive TST which imply that children in this study did not meet the criteria established in the country and therefore did not receive treatment for LTBI.

Subsequently, in the same city of Colombia in 2012, a study was conducted on children under five years living with patients with bacillary pulmonary TB. It was found that 28.6% children who were contacted by telephone had been adequately studied (with chest X-ray and TST); for children who attended clinical evaluation, the prevalence of active TB was 7.9%, and only 19.4% received treatment for LTBI. Given that most of the household contact children were not adequately studied and therefore did not receive the indicated treatment, there was no significant impact on the decrease in the incidence of TB in these children with respect to the cohort study conducted between 2005 and 2009 [44].

Considering those findings, in 2015, a cohort study in Medellín, Bello and Itagüí, Colombia was started, with a before-after design and a two-year follow-up of children under five years of age who were household contacts of pulmonary TB patients to analyze the effectiveness of LTBI treatment with nine months of isoniazid as recommended by the national TB program, by comparison with children from a 2005–2009 cohort who did not receive treatment. The treatment cascade showed that from 329 children who met the inclusion criteria, 74% were assessed, 66% started treatment, 45% received LTBI treatment for at least six months, and 23.1% for nine months. The qualitative approach allows us to understand the complexity that underlies the effectiveness of LTBI treatment. This can be summarized in three categories: daily life and conditions of possibility for children exposed to TB and their families (including forced migration); the Colombian health system does not guarantee real access to TB diagnosis and treatment; and perceptions and social constructions around TB (TB remains as a stigma) [24]. Considering these findings, the research team proposed the present protocol study addressing some of the issues aiming to tackle the treatment completion barriers by an integrated care strategy for children exposed to TB.

This strategy should allow children to be evaluated at a single site without having to go through different institutions and levels of authorization that hinder and delay both diagnosis and treatment. Comprehensive care should include both general practitioners and specialists, such as child infectious disease specialists, as well as other disciplines, like nutrition, psychology, and social work. At the same time, access to diagnostic aids should be available. In addition, the possibility to evaluate the effectiveness of the strategy, along with a comparison with different available treatments on the market and recommended in the new guidelines, such as rifampicin for four months.

After conducting this study, we expect to support the WHO recommendations regarding more comprehensive strategies to assess, treat and follow children exposed to TB, contributing to the End TB strategy goals.

## Trial status

Protocol version September 28, 2020. Recruitment began on July 26, 2021. Treatment and follow-up are ongoing.

## Supplementary Information

**Additional file 1.**

## Data Availability

The datasets generated and/or analyzed during the current study are not publicly available due to the ongoing study but are available from the corresponding author at reasonable request.

## Abbreviations

CIB: *Coporación para Investigaciones Biológicas*
HIV: Human immunodeficiency virus
IGRA: Interferon Gamma Release Assay
LTBI: Latent tuberculosis infection
QFT Plus: QuantiFERON®-TB Gold Plus
SPIRIT: Recommendations for Interventional Trials
TB: Tuberculosis
TST: Tuberculin skin test
WHO: World Health Organization
